# Mechanisms of small cell lung cancer metastasis

**DOI:** 10.15252/emmm.202013122

**Published:** 2020-12-09

**Authors:** Julie Ko, Monte M Winslow, Julien Sage

**Affiliations:** ^1^ Department of Pediatrics Stanford University Stanford CA USA; ^2^ Department of Genetics Stanford University Stanford CA USA; ^3^ Department of Pathology Stanford University Stanford CA USA

**Keywords:** lung cancer, metastasis, NFIB, SCLC, tumor heterogeneity, Cancer, Respiratory System

## Abstract

Metastasis is a major cause of morbidity and mortality in cancer patients. However, the molecular and cellular mechanisms underlying the ability of cancer cells to metastasize remain relatively poorly understood. Among all solid tumors, small cell lung cancer (SCLC) has remarkable metastatic proclivity, with a majority of patients diagnosed with metastatic disease. Our understanding of SCLC metastasis has been hampered for many years by the paucity of material from primary tumors and metastases, as well as the lack of faithful pre‐clinical models. Here, we review recent advances that are helping circumvent these limitations. These advances include methods that employ circulating tumor cells from the blood of SCLC patients and the development of diverse genetically engineered mouse models of metastatic SCLC. New insights into the cellular mechanisms of SCLC metastasis include observations of cell fate changes associated with increased metastatic ability. Ongoing studies on cell migration and organ tropism promise to expand our understanding of SCLC metastasis. Ultimately, a better molecular understanding of metastatic phenotypes may be translated into new therapeutic options to limit metastatic spread and treat metastatic SCLC.

## Graphical abstract

Small cell lung cancer (SCLC) is highly metastatic, but rare resections of SCLC tumors and metastases have hampered our understanding of SCLC progression. This review offers a comprehensive overview of recent advances on models and mechanisms of SCLC metastasis.

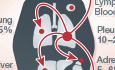

**Glossary**
Circulating tumor cells (CTCs): Cancer cells that have left a tumor and circulate in the bloodstream.
Epithelial‐to‐mesenchymal transition (EMT): The process by which epithelial cells acquire mesenchymal features. EMT in cancer is often associated with increased metastatic potential.
Immune checkpoint inhibitors: Inhibitors of negative regulators of immune reactions. Upon inhibition of these immune checkpoints, the anti‐cancer activity of immune cells can be enhanced.
Macro‐metastasis: A metastasis that can be detected without a microscope, often exceeding 2 mm in diameter in a cancer patient.
Metastatic cascade: The series of steps by which cancer cells leave the primary tumor and form metastases.
Micro‐metastasis: A small metastasis that can be best detected microscopically.
Neuroendocrine tumors: Tumors in which cancer cells have traits similar to those of both nerve cells and hormone‐producing cells.
Pleural effusion: Excess fluid around the lung. The growth of lung cancer cells outside of the lungs often generates pleural effusions.
Tumor organoids: Cellular clusters derived from tumors that mimic the three‐dimensional *in vivo* characteristics of tumors.
Vasculogenic mimicry (VM): The process by which cancer cells acquire features of vascular cells and organize themselves into blood vessel‐like structures.

## Introduction

### Metastases represent a major clinical problem

Cancer is a leading cause of death, resulting in close to 9 million deaths worldwide every year. Tissue destructive macro‐metastases disrupt organ function and are a major source of morbidity and lethality for almost all solid tumor types (reviewed in (Gupta & Massagué, 2006; Hanahan & Weinberg, 2011; Dillekås *et al*, 2019)).

Cells progressing through the metastatic cascade must employ a series of diverse cellular processes, from local invasion and intravasation into the bloodstream, survival in circulation, and extravasation, to colonization and growth in new organ environments. These different steps are associated with different molecular programs that regulate migration, survival, and proliferation of cancer cells (reviewed in (Stracke & Liotta, 1992; Nguyen & Massagué, 2007; Lambert *et al*, 2017)). Despite this conceptual framework, the molecular and cellular mechanisms that allow an initially benign expansion of cells to gain the ability to overcome all of the hurdles of the metastatic process remain poorly understood. In part, this is due to the paucity of metastatic samples that can be obtained from patients, especially those with paired primary tumor samples (reviewed in (Nguyen *et al*, 2009; Lambert *et al*, 2017)). In addition, cell lines grown in culture may fail to accurately recapitulate metastatic behavior and may not faithfully model the complex processes involved in each step of metastatic progression *in vivo*. Although different cancers share the same general steps of metastatic progression, the diversity of molecular programs across (and within) cancer types necessitates the analysis of cancer type specific molecular and cellular programs that contribute to metastasis.

### Small cell lung cancer is highly metastatic

**Figure 1:**
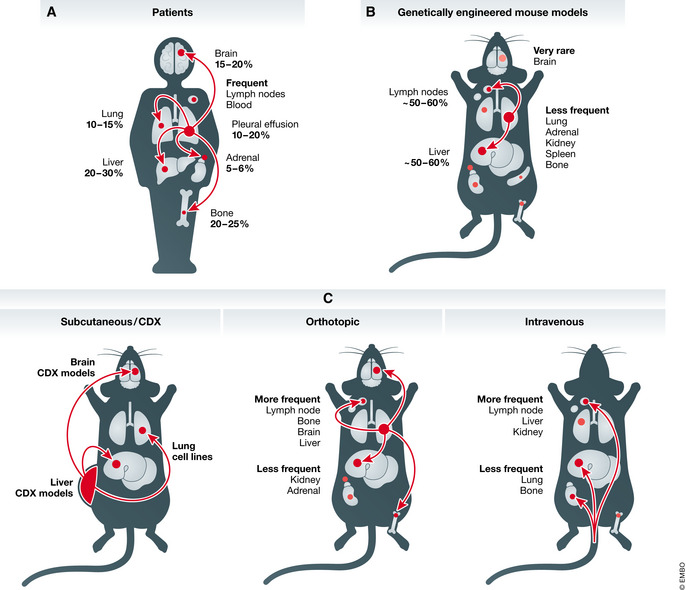
Metastatic ability of SCLC Schematic representation of common metastatic sites in SCLC patients (A), genetically engineered mouse models (B), and pre‐clinical human models in mice (C). CDX, circulating tumor cell‐derived xenograft. See text for details.

Small cell lung cancer (SCLC) is a highly metastatic and recalcitrant carcinoma. While worldwide data for SCLC are not available, it is estimated that SCLC accounts for ~ 15% of lung cancers and causes more than 200,000 deaths per year (reviewed in (Gazdar *et al*, 2017; Sabari *et al*, 2017)). Patients with localized SCLC confined within the lung sometimes undergo surgical resection to remove their primary tumor, and this is associated with improved survival (Weksler *et al*, 2012; Barnes *et al*, 2017; Wang *et al*, 2020b). However, close to 70% of SCLC patients already have metastatic disease at diagnosis, with macro‐metastases commonly found in the lymph nodes, brain, liver, and bones (Fig 1). In these patients, surgical resection was shown decades ago to be less effective than radiotherapy or chemotherapy and is thus rarely performed (Miller *et al*, 1969; Fox & Scadding, 1973). The median survival for SCLC patients is only 7–12 months after diagnosis (de Castro Carpeño *et al*, 2019; Chung *et al*, 2020) and reviewed in (Byers & Rudin, 2015)). Over the past 30 years, the only major change to standard chemotherapy and radiation treatments has been the recent approval of immune checkpoint inhibitors, which extend survival by several months (Antonia *et al*, 2016; Horn *et al*, 2018; Ready *et al*, 2020; Rudin *et al*, 2020). Prophylactic cranial irradiation can reduce the emergence of brain metastases; however, this treatment modality does not consistently improve disease‐free and overall survival (Aupérin *et al*, 1999; Slotman *et al*, 2007; Takahashi *et al*, 2017). Thus, identifying novel ways to limit metastatic spread and target metastases is critical for the development of new therapies to treat SCLC (Sabari *et al*, 2017).

## Models of SCLC metastasis

### Circulating tumor cells as a new tool to investigate human SCLC metastasis

Small cell lung cancer cell lines generated from pleural effusions and metastases from patients with advanced disease are commonly used for *in vitro* analysis, including studies of cell adhesion and cell migration (Carney *et al*, 1985; Gazdar *et al*, 1985), and *in vivo* analysis in xenografts (subcutaneous, intravenous, and orthotopic in the lungs; Table 1 and Fig 1). However, it is difficult to know whether these cell lines have retained the relevant metastatic features of the cells from which they originated. Patient‐derived xenografts (PDXs) generated from resected tumors complement cell line‐based studies (Gardner *et al*, 2017; Drapkin *et al*, 2018). These models are often used at early passages and are more likely to retain the features of their tumor of origin. However, they are almost invariably generated from primary tumors (as these are the tumors that are most often resected from patients) and they do not readily metastasize to distant sites when grown subcutaneously. The subcutaneous growth of PDXs also fails to recapitulate the microenvironment of the lungs or metastatic sites, and studies based on orthotopic injections are rare (Weiskopf *et al*, 2016). Furthermore, PDXs are by necessity grown in the absence of adaptive immune cells, which is becoming an even greater limitation as T cell‐based immunotherapies are gaining clinical traction for treating SCLC.

**Table 1:** Metastatic ability of human SCLC pre‐clinical models.

Name	Site of origin	Model (reference)	Site of metastasis							
			Lymph node	Bone	Brain	Liver	Kidney	Lung	Adrenal Gland	CTCs
MDA‐SC39	CTCs	CDX model—grown subcutaneously (Stewart *et al*, 2020)			✓					
CDX14P	CTCs	CDX model—grown subcutaneously (Simpson *et al*, 2020)			✓	✓				✓
CDX17	CTCs	CDX model—grown subcutaneously (Simpson *et al*, 2020)				✓				✓
DMS273	Pleural effusion	Cell line—orthotopic transplant into lung (Sakamoto *et al*, 2015)	✓	✓	✓	✓	✓		✓	
NCI‐H187	Pleural effusion	Cell line—orthotopic transplant into lung (Jacoby *et al*, 2010)	✓	✓	✓	✓				
NCI‐H250	Brain metastasis	Cell line—carotid artery injection (Li *et al*, 2013)			✓					
NCI‐H446	Pleural effusion	Cell line—tail vein injection (Wang *et al*, 2011)						✓		
NCI‐H69	Pleural effusion	Cell line—lung metastasis from subcutaneous transplant (Sodeur *et al*, 2009) Cell line—lymph node, liver, kidney metastasis from tail vein injection (Miki *et al*, 2000)	✓			✓	✓	✓		
NCI‐H82	Pleural effusion	Cell line—subcutaneous tumor (Sodeur *et al*, 2009)						✓		
SBC‐3	Bone marrow metastasis	Cell line—tail vein injection (Miki *et al*, 2000)	✓			✓	✓			
SBC‐5	Pleural effusion	Cell line—tail vein injection (Miki *et al*, 2000)	✓	✓		✓	✓			

CTC, circulating tumor cells; CDX, CTC‐derived xenograft.

New models have recently been developed from SCLC circulating tumor cells (CTCs). A seminal study found that SCLC patients have uniquely high numbers of CTCs (Hou *et al*, 2012). CTC numbers in patients correlate with disease stage and survival (Aggarwal *et al*, 2017; Tay *et al*, 2019), and genomic alterations found in CTCs reflect those commonly found in SCLC tumors (Carter *et al*, 2017). The recent development of CTC‐derived xenograft models (CDX models) and primary cell cultures raises new possibilities for the study of SCLC metastasis (Hodgkinson *et al*, 2014; Carter *et al*, 2017; Drapkin *et al*, 2018; Lallo *et al*, 2019). However, it remains unclear how closely CDX models reflect SCLC metastases growing in different organ environments. For instance, the CTCs that generate CDXs may or may not reflect the cells that seed metastases in patients. CTCs may also originate from metastases rather than primary tumors, thus representing a later stage of metastatic propagation. Nonetheless, CTCs and CDX models represent a paradigm shift in our ability to study SCLC metastasis: While published human SCLC cell lines or PDX models have not been reported to efficiently metastasize when implanted into mice, recent CDX models have been shown to have the potential to seed metastases in the liver and the brain of recipient mice (Table 1 and Fig 1) (Simpson *et al*, 2020; Stewart *et al*, 2020). It is likely that an increasing number of studies will employ CDX models to investigate the cellular mechanisms of human SCLC metastasis.

### Genetically engineered mouse models of metastatic SCLC

The *RB1* and *TP53* tumor suppressors are nearly ubiquitously inactivated in human SCLC (Harbour *et al*, 1988; Takahashi *et al*, 1989; George *et al*, 2015). This observation led to the development of the first genetically engineered mouse model of SCLC. In this *RP* model, floxed alleles of *Rb* (*Rb1*) and *p53* (*Trp53*) enable the homozygous inactivation of these genes in lung epithelial cells of adult mice using adenoviral vectors expressing Cre recombinase (Ad‐Cre) (Meuwissen *et al*, 2003). SCLC tumors in this model represent the predominant subtype of SCLC, characterized by expression of neuroendocrine markers and high levels of the transcription factor ASCL1 (“SCLC‐A” subtype) (Rudin *et al*, 2019). Approximately half of the mice develop metastases to the lymph nodes, liver, and a variety of other organs including the spleen and the kidneys (Meuwissen *et al*, 2003; McFadden *et al*, 2014) (Fig 1). Subsequent models have included the inactivation of the *Pten*, *Crebbp*, or *Rbl2* (also known as *p130*) tumor suppressors in the context of the *RP* model. Inactivation of these genes, which are each recurrently mutated in human SCLC (George *et al*, 2015), increased primary tumor number and tumor growth. Similar to the *RP* model, 50–60% of these different triple‐mutant mice develop metastases, especially in the liver (Schaffer *et al*, 2010; Cui *et al*, 2014; Gazdar *et al*, 2015; Jia *et al*, 2018).

In addition to the coincident inactivation of additional tumor suppressor genes, members of the MYC family of oncogenes are frequently amplified in human SCLC (George *et al*, 2015). A mouse model incorporating expression of a constitutively active form of MYC coincident with *Rb* and *p53* deletion (*Rb/p53/Myc* or *RPM* model) shows rapid growth of SCLC tumors that are highly metastatic and metastasize within weeks of initiation (Mollaoglu *et al*, 2017; Dammert *et al*, 2019). This model represents the second most common SCLC subtype, characterized by high expression of the transcription factor NEUROD1 (“SCLC‐N” subtype) (Rudin *et al*, 2019). Interestingly, mouse models for two rarer subtypes of SCLC (SCLC‐Y and SCLC‐P, with high levels of YAP and POU2F3, respectively) have not yet been described (Rudin *et al*, 2019). With the exception of some studies on the *Rb/p53/p130* mutant mouse model of SCLC (also known as *RPR2* or *TKO* for triple knock‐out) (see below), metastatic programs of the *Pten* or *Crebbp* mutant SCLC‐A subtype or the *RPM* SCLC‐N subtype have not yet been described. As mouse models for specific SCLC subtypes and genotypes are developed, molecular and functional characterization of the programs that drive their metastatic ability will provide further insights into how metastases differ across this heterogeneous cancer. Investigating how the different genetic alterations that drive initiation and growth affect metastatic programs will eventually help identify specific metastatic programs that can be targeted in a more precise manner.

Genetically engineered mouse models of SCLC have several beneficial features in studies of metastasis, including the growth of primary tumors and metastases in an immunocompetent host and in physiologically relevant microenvironmental contexts. Early‐ and late‐stage primary tumors as well as metastases can be isolated from these mouse models for cellular and molecular analyses. Incorporating fluorescent Cre‐reporters in these models has allowed cancer cells to be readily distinguished from non‐cancer cells, which can help tease out cancer cell intrinsic and extrinsic mechanisms. However, mouse models also have limitations. While brain metastases are frequent in SCLC patients, they are very rare in mouse models, as only one mouse with SCLC has been described with a brain metastasis (Meuwissen *et al*, 2003; Lukas *et al*, 2017). In addition, mouse SCLCs have fewer genomic alterations than human tumors, the development of which is associated with mutations caused by tobacco smoking (McFadden *et al*, 2014; George *et al*, 2015). This low mutation burden in mouse tumors may limit the evolution of mouse tumors, reduce immunogenicity, and potentially impact how well metastases in these models reflect human biology. Nevertheless, mouse models have proven valuable in identifying molecular and cellular mechanisms of SCLC metastasis that have been validated in human tumors.

While the culture of CTCs and other primary SCLC cells, the development of CDX and PDX models, and the genetically engineered mouse models continue to improved our ability to investigate the biology of SCLC and molecular and cellular mechanisms of metastasis, additional pre‐clinical models could be developed. For instance, new protocols to grow organoids derived from primary lung tumors, including neuroendocrine tumors (Sachs *et al*, 2019; Kawasaki *et al*, 2020), may provide new ways to investigate cell–cell interactions important for the dissemination of SCLC. It is also possible that in the future other animal models, such as rats, ferrets, rabbits, or pigs, may be used to generate pre‐clinical models of SCLC.

## Molecular mechanisms of SCLC metastasis

### Upregulation of NFIB during acquisition of metastatic ability in SCLC

Accumulating evidence suggests that metastasis can occur early during the tumorigenic process, including the dissemination of cancer cells from early lesions (Hosseini *et al*, 2016). Recent modeling of metastasis from human colorectal cancer based on DNA sequencing also suggests that genetic alterations that drive metastasis are acquired early during tumor evolution (Hu *et al*, 2019). In SCLC, the high mutational burden induced by cigarette smoking, fast tumor growth, and high percentage of patients diagnosed with metastatic disease support a model in which primary tumors could be inherently metastatic. However, the analysis of clonal heterogeneity during SCLC progression in the *RP* mouse model suggested strong bottlenecks during metastatic progression (McFadden *et al*, 2014). The analysis of primary tumors and metastases in the *RPR2* mouse model using a multicolor lineage‐tracing reporter similarly showed that metastases were typically seeded by only 1 or 2 out of more than 50 primary tumors (Yang *et al*, 2018). Furthermore, some *RPR2* mice with large primary tumors had no metastases of any kind, suggesting that these tumors do not have metastatic potential. Even after 6–7 months of tumor growth, some of these mice had no detectable disseminated cancer cells in their pleural cavity indicating that these tumors had also not overcome this early hurdle of the metastatic process (Yang *et al*, 2018). These data from mouse models strongly suggest that the metastatic potential of SCLC is acquired during tumor progression.

Molecular analyses of SCLC cells isolated from *RPR2* mice uncovered large‐scale changes in chromatin accessibility and differences in gene expression programs between primary tumors and metastases (Denny *et al*, 2016). Through these analyses and functional validation assays, increased expression of the NFIB transcription factor was identified as a pro‐metastatic switch. Unlike early tumors, which express low levels of NFIB, some larger primary tumors and the vast majority of metastases in *RPR2* and *RP* mice express high levels of NFIB (Dooley *et al*, 2011; Denny *et al*, 2016; Böttger *et al*, 2019). Overexpression of NFIB is oncogenic in *RP* mice and some SCLC cell lines, and it promotes metastasis to the liver and other organs (Dooley *et al*, 2011; Denny *et al*, 2016; Semenova *et al*, 2016; Wu *et al*, 2016; Böttger *et al*, 2019). Furthermore, NFIB expression is associated with several gene expression programs related to metastasis, including cell migration (Denny *et al*, 2016; Wu *et al*, 2016). In human SCLC, NFIB is highly expressed in ~ 50% of tumors and metastases, and high expression correlates with worse patient survival (Dooley *et al*, 2011; Denny *et al*, 2016; Semenova *et al*, 2016). However, while high expression of NFIB in mouse SCLC is often linked to genomic amplification of the *Nfib* gene, this is unlikely to be the only mechanism that drives NFIB expression in these tumors. Furthermore, the *NFIB* gene is not very frequently amplified in human SCLCs, and the range of mechanisms by which NFIB levels are upregulated remain unknown. One possible mechanism may involve the transcription factor MYC, which can drive high NFIB levels, as seen in *RPM* mice (Mollaoglu *et al*, 2017). While expression of the MYC family members MYC, MYCL, and MYCN is frequently upregulated in human SCLC, tumors in *RP* mice with overexpression of *Mycl* do not have high levels of NFIB, suggesting this may be a mechanism specific to MYC (Sos *et al*, 2012; Semenova *et al*, 2016). However, the role of NFIB in the metastasis of MYC‐high SCLC is not known yet.

Together, the identification of the transcription factor NFIB in mouse models and its validation in human tumors indicates that acquiring high levels of NFIB by gene amplification and/or other mechanisms contribute to the acquisition of metastatic ability by SCLC. The upregulation of pro‐metastatic gene expression programs in SCLC cells represents an opportunity to better understand the biology of metastatic SCLC but also identifies molecular handles with which to pursue specific targeting of SCLC metastases.

### NFIB‐independent mechanisms of metastatic progression

**Figure 2:**
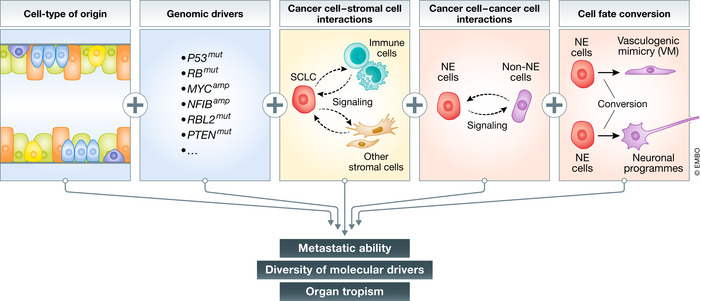
Mechanisms of SCLC metastasis The metastatic ability of SCLC is regulated by a number of factors, including the cell type from which tumors originate, the genetic make‐up of each tumor, interactions between cancer cells and other cells in the tumor microenvironment, and epigenetic factors regulating cell fate and intratumoral heterogeneity. See text for details.

While NFIB upregulation may represent a key molecular mechanism of metastasis in a subset of human SCLC, other mechanisms certainly contribute to the metastatic ability of SCLC. Notably, NFIB‐driven metastasis was identified in the *RPR2* model in which tumors were initiated through transduction of lung epithelial cells with Adenoviral‐CMV‐Cre (Denny *et al*, 2016). Because the transduced cells encompass several cell types, the identity of the cell type that gives rise to SCLC in this context is still unknown. However, when tumors were initiated in the same *RPR2* model specifically from pulmonary neuroendocrine cells (Ferone et al, 2020) that express the neuroendocrine marker CGRP (using Adeno‐CGRP‐Cre‐mediated deletion of *Rb/p53/p130*), the resulting metastatic SCLC did not upregulate NFIB (Yang *et al*, 2018). These observations show that the cell type of origin can have a profound influence on the molecular mechanisms by which metastatic ability is eventually gained (Fig 2). Adeno‐CGRP‐Cre‐initiated tumors in *RPR2* mice will provide a model for future work investigating NFIB‐independent mechanisms of metastasis in SCLC (Yang *et al*, 2018).

More generally, accumulating evidence suggests that both the initiating genetic alterations and the cell type of origin influence the development of SCLC tumors and their phenotypes (Mollaoglu *et al*, 2017; Huang *et al*, 2018; Yang *et al*, 2018; Böttger *et al*, 2019; Ireland *et al*, 2020) (reviewed in (Ferone *et al*, 2020)). The extent of interplay between cell type of origin and genetics remains largely unknown for any cancer type. It is possible that different combinations of genetic alterations and cell types of origin will give rise to tumors that gain metastatic potential via distinct molecular and cellular mechanisms. A better understanding of the mechanisms used by pre‐metastatic SCLC cells to become metastatic, as well as the identification of markers for these different mechanisms, will contribute to personalized treatment for different subtypes of metastatic SCLC.

### Cell–cell interactions in primary tumors can promote SCLC metastasis

In addition to cancer cell intrinsic, genetic, and transcriptional mechanisms that contribute to metastatic progression, mechanisms that involve cell–cell interactions between different populations of SCLC cells may also influence metastatic ability (Fig 2). Accumulating evidence indicates that SCLC tumors are often composed of neoplastic cells with distinct features including neuroendocrine (NE) and less/non‐neuroendocrine (non‐NE) cells (Shue *et al*, 2018). Interestingly, this intratumoral heterogeneity has been suggested to be critical for the early steps of metastasis. When NE‐only and non‐NE‐only mesenchymal‐like cell lines (derived from single cells from tumor in an *RP* mouse) were transplanted subcutaneously, they engrafted and grew tumors, but did not generate metastases. However, subcutaneous tumors generated from a mixed population of NE and non‐NE SCLC cells were uniquely capable of generating metastasis from the NE cells (Calbo *et al*, 2011). When transplanted intravenously, NE or non‐NE cells gave rise to a similar liver metastasis burden as mixed populations, suggesting that NE/non‐NE interactions affect steps prior to and/or including intravasation (Kwon *et al*, 2015). One mechanism through which this heterogeneity may confer metastatic ability is through the secretion of FGF2 by non‐NE cells, which induces expression of the pro‐metastatic transcription factor PEA3 in the NE cells (Kwon *et al*, 2015).

Vasculogenic mimicry (VM) is a process through which cancer cells acquire features of endothelial cells, enabling *de novo* formation of tumor‐derived vascular networks. VM has been associated with tumor progression and metastasis in other cancers, such as breast cancer (Wagenblast *et al*, 2015), and has been described in human SCLC tumors and CDX models (Williamson *et al*, 2016). VM may promote metastasis in multiple ways in addition to increased angiogenesis and blood flow, which aid in intravasation into the vascular system. For instance, a small subpopulation of SCLC CTCs expresses the VM marker VE‐cadherin (Williamson *et al*, 2016). One potential model is that interactions between VM SCLC cells and non‐VM SCLC cells may continue once non‐VM SCLC cells have left the primary tumor and are circulating in the blood, possibly promoting their survival. Furthermore, intra‐vital imaging has found that SCLC cells roll along vessel walls, similar to leukocytes (Heidemann *et al*, 2014), and it is thus possible that non‐VM SCLC cells may interact similarly with VM SCLC cells (and normal endothelial cells), which may contribute to metastasis. These ideas should be more thoroughly tested in appropriate pre‐clinical models.

Another distinct population of non‐NE SCLC cells can be generated by activation of Notch signaling in the tumor microenvironment, and this population can promote the growth of NE SCLC cells, including following chemotherapy treatment (Lim *et al*, 2017). However, it is unclear whether Notch‐positive non‐NE SCLC cells also promote the metastatic ability and/or metastatic outgrowth of their NE counterparts. Identifying additional cellular heterogeneity and cell–cell interactions will provide new opportunities to understand pro‐metastatic biology in SCLC.

### Molecular mechanisms promoting the migration of SCLC cells

Early work showed that attachment to the extracellular matrix (ECM) via β1‐integrin could activate PI3K signaling and promote the survival of SCLC cells following DNA damage (Feldman *et al*, 1991; Oshita *et al*, 2004). Recent studies have further identified a role for β1‐integrin in the migration and metastatic ability of SCLC cells in part via FAK (focal adhesion kinase) signaling (Zhao *et al*, 2019). Additionally, the cell adhesion molecule E‐cadherin (CDH1) is highly expressed in early lesions, then downregulated in metastatic tumors (Semenova *et al*, 2016; Böttger *et al*, 2019). Low levels of E‐cadherin also correlate with shorter survival in SCLC patients (Chang *et al*, 2012), but few molecular studies have specifically investigated the role of E‐cadherin in the migration and metastasis of SCLC cells.

The adhesion of SCLC cells to β1‐integrin ligands is also promoted by the interaction between the chemokine CXCL12 and its receptor CXCR4, which can provide protection from apoptosis (Hartmann *et al*, 2005). The interaction between CXCL12 and CXCR4 has also been implicated in the migration of SCLC cells (Burger *et al*, 2003; Ma *et al*, 2015; Taromi *et al*, 2016). CXCR4 expression is upregulated in SCLC, and high expression of CXCR4 in patients correlates with bone metastases and poor survival (Li *et al*, 2015). Inhibiting CXCR4 signaling, either by downregulation of CXCR4 or treatment with CXCR4 inhibitors, suppresses formation of metastases in an *in vivo* transplant model (Ma *et al*, 2015; Taromi *et al*, 2016). CXCR4 antagonists have been developed (Mousavi, 2020), and some activity has been detected in pre‐clinical models (Taromi *et al*, 2016); however, the relationship between CXCR4 and SCLC metastases has yet to be validated in models beyond *in vitro* and transplant models. Thus, it remains unknown whether their application could block or slow metastatic propagation in SCLC in patients. The role of integrins and other adhesion molecules in the interactions between different subtypes of SCLC cells (e.g., NE and non‐NE cells) has not yet been studied. Such interactions between SCLC cells could be particularly important for clusters of CTCs found in the blood of patients (Hou *et al*, 2012), possibly promoting the survival or extravasation of these cancer cells.

Delta‐like ligand 3 (DLL3) is an atypical ligand for NOTCH receptors that is expressed on SCLC stem cells (Saunders *et al*, 2015), circulating tumor cells (Messaritakis *et al*, 2019; Obermayr *et al*, 2019), and metastases (Sharma *et al*, 2017). DLL3 may play a role in the migration of SCLC cells (Furuta *et al*, 2019; Huang *et al*, 2019). DLL3 is an emerging target in SCLC (Morgensztern *et al*, 2019; Hipp *et al*, 2020) and it will be interesting in the near future to understand whether targeting this molecule can help block the formation of new metastases or slow the growth of established ones.

Epithelial–mesenchymal transition (EMT) is an evolutionarily conserved program that has been implicated in enhancing mobility, invasion, and resistance to cell death during metastasis (Chaffer *et al*, 2016). Features of EMT have been observed in SCLC where they may be linked to resistance to chemotherapy (Stewart *et al*, 2017, 2020). Notch signaling has also been linked to EMT in SCLC, which may provide a mechanism for SCLC cells to control their epithelial or mesenchymal features (Hassan *et al*, 2014). As discussed above, lower levels of E‐cadherin in SCLC metastases suggest that metastatic tumors may have more mesenchymal features. Overall, however, the role of EMT in SCLC metastasis remains unclear and future studies will need to examine in more detail the transcriptional programs associated with EMT in SCLC cells in different contexts and how EMT may contribute to various aspects of SCLC metastasis.

It has long been recognized that SCLC cells can express neuronal markers (reviewed in (Onganer *et al*, 2005)). Expression of neuronal markers such as neuron‐specific enolase can serve as a marker for more aggressive disease in SCLC patients (Akoun *et al*, 1985; Jørgensen *et al*, 1996; Kanemoto *et al*, 2006). Recent work in mouse models has found that SCLC cells increase their expression of neuronal gene programs during metastatic progression. Some of these gene expression programs are under the control of NFIB (Steele‐Perkins *et al*, 2005; Denny *et al*, 2016; Semenova *et al*, 2016), but expression of neuronal differentiation markers is also found in metastases in which NFIB expression is not upregulated (Yang *et al*, 2018). This suggests that this neuroendocrine‐to‐neuronal transition is a common feature of SCLC metastatic progression (Fig 2). While these neuronal programs may affect multiple processes in SCLC cells, one mechanism by which neuronal differentiation may promote SCLC metastasis is through the generation of long protrusions that resemble axons. These axon‐like protrusions, which have been observed in culture and *in vivo* in both mouse and human models, facilitate the migration of SCLC cells in culture in a manner similar to neuroblast migration (Yang *et al*, 2019). How these axon‐like protrusions contribute to other aspects of the metastatic cascade *in vivo* remains unknown. Knock‐down of several diverse genes associated with the formation of these protrusions in SCLC cell lines did not affect subcutaneous tumor growth, but decreased the number of liver metastases after intravenous transplantation (Yang *et al*, 2019). These results suggest that these protrusions aid in metastatic seeding. NFIB expression does not seem to be sufficient for the formation of these protrusions (Yang *et al*, 2019), and the molecular and cellular mechanisms that control their formation remain only partly understood. Finally, given that some SCLC tumors originate from pulmonary neuroendocrine cells, it could be of interest to compare the motility and migration properties of SCLC cells to those of normal developing lung neuroendocrine cells that “slither” during development (Kuo & Krasnow, 2015). What cellular inputs control these cell state transitions, how these new cellular phenotypes drive migration and different steps of the metastatic cascade, and how they can be reverted or manipulated will be important areas of future investigation.

### Organ tropism

Small cell lung cancer tumors commonly metastasize to the brain, liver, bones, and distant lymph nodes, but also metastasize at lower frequencies in many other organs (Froudarakis, 2012; Nakazawa *et al*, 2012; Ryu *et al*, 2016; Bütof *et al*, 2017; Wang *et al*, 2020a) (Fig 1). Little is known about the molecular and cellular mechanisms that allow SCLC cells to home to, seed, and grow into micro‐ and macro‐metastases in specific organ environments. As noted above, tumors in genetically engineered mouse models form extrapulmonary metastases in lymph nodes and similar organs to those affected in patients, except for the brain (Meuwissen *et al*, 2003) (Fig 1). One simple explanation for this discrepancy between mice and humans is that morbidity associated with tumor burden in the lung and the liver in mice with SCLC requires euthanasia before brain metastases have the time to form. However, differences in the blood–brain barrier between mice and humans (Aday *et al*, 2016; O’Brown *et al*, 2018) and other factors should be considered. It is interesting to note that intravenous injection of mouse SCLC cells does not result in the development of lung metastases, the first major organ that the cancer cells transit through, but nearly exclusively results in liver metastases (e.g., (Kwon *et al*, 2015; Denny *et al*, 2016)), an observation which remains unexplained.

Interestingly, the genetic alterations in SCLC may also influence the locations of metastases. Notably, overexpression of NFIB in the *RP* model increased kidney and bone metastases (Semenova *et al*, 2016), as well as bronchiolar, but not alveolar, intrapulmonary metastases (Böttger *et al*, 2019). Elevated NFIB levels could simply increase general metastatic seeding ability or tumor growth, but it is also possible that NFIB upregulation provides an additional advantage for growth in those specific microenvironments. For instance, NFIB is essential for lung and brain development in mice (Steele‐Perkins *et al*, 2005); thus, NFIB could modulate conserved targets that influence how SCLC cells interact with specific microenvironments in the lungs and brain.

Brain metastases occur in around 15–20% of SCLC patients at the time of diagnosis (Nakazawa *et al*, 2012). The incidence of SCLC brain metastases increases to almost 80% during the course of the disease and is so common that prophylactic brain irradiation protocols have been developed (Takahashi *et al*, 2017; Yin *et al*, 2019). Previous studies of SCLC brain metastases have described upregulation of angiogenesis‐related factors, with some, such as PDGFRB and ANGPTL4, specifically upregulated in SCLC brain metastases compared to those from NSCLC and melanoma (Ilhan‐Mutlu *et al*, 2016). Angiogenic factors such as placental growth factor (PLGF) and vascular endothelial growth factor receptor‐1 (VEGFR‐1) have also been implicated in transendothelial migration across the blood–brain barrier. SCLC cells secrete PLGF which binds to VEGFR‐1 on brain microvascular endothelial cells and breaks down tight junctions in the blood–brain barrier (Li *et al*, 2006, 2013). Expression of PLGF in brain metastases is higher than in primary lung tumors from patients without brain metastases, which suggests that it could increase SCLC proclivity for brain metastasis (Li *et al*, 2013). As noted above, recent CDX models show metastatic potential to the brain in recipient mice (Table 1) (Simpson *et al*, 2020; Stewart *et al*, 2020); thus, these models may represent a new platform with which to investigate the molecular and cellular mechanisms of SCLC brain metastasis.

Another common site for SCLC metastasis is the bone, with around 20% of SCLC patients developing bone metastases (Nakazawa *et al*, 2012). Some factors involved in migration (e.g., β3‐integrin (Li *et al*, 2012) and CXCR4 (Ma *et al*, 2015)) have been found to affect SCLC metastatic ability to the bone, but otherwise, not much is known about SCLC bone metastasis. CXCR4 in particular has been implicated in bone metastases from other cancer types (e.g., (Geminder *et al*, 2001; Hung *et al*, 2014)) and could be especially relevant due to its role in hematopoietic stem cell homing to the bone marrow. As with the increase in bone metastases in the *RP* mouse model with overexpression of NFIB (Semenova *et al*, 2016), whether these factors have bone‐specific roles or are generally pro‐metastatic has yet to be determined. Overall, our understanding of organ‐specific mechanisms of SCLC metastasis remains extremely limited even though studies of this phenomenon are certain to uncover interesting and important new biology.

## Conclusion

### Clinical benefits of therapies against metastases in SCLC

Small cell lung cancer is an aggressively metastatic cancer. While an increase in knowledge of the genetic drivers of lung adenocarcinoma has enabled the development of new treatments that have extended survival and decreased mortality, the decrease in mortality from SCLC has been strictly driven by decreased incidence (Howlader *et al*, 2020). Consequently, there remains a tremendous need to understand the biology of metastatic SCLC and translate that knowledge into better therapies. Not only is there a need to better treat metastatic SCLC, but as early cancer detection methods improve, more patients will be diagnosed with SCLC before developing metastatic disease. Thus, the development of effective therapies that prevent metastatic spread will become more clinically valuable. Recent advances in modeling metastatic SCLC have uncovered genetic and epigenetic changes as well as cell fate transitions that promote metastatic potential and multi‐organ metastatic growth. The identification of these changes could be a first step in the development of novel targeted therapies to treat and prevent metastatic SCLC.

### Cell type of origin, genomic alterations, and cell–cell interactions influence SCLC progression and therapy responses

Differences in treatment responses between different models of SCLC are becoming increasingly apparent (Ferone *et al*, 2020) perhaps beginning to mirror the diversity of patient responses in the clinic. Research on models of specific SCLC subtypes has found differential sensitivity to certain treatments (e.g., (Mollaoglu *et al*, 2017; Böttger *et al*, 2019)). Interestingly, some evidence suggests that different SCLC subtypes could also represent different stages of tumor progression, rather than entirely distinct tumor types (Ireland *et al*, 2020). Furthermore, although the connections between subtypes and cell types of origin have yet to be defined, one study has suggested that SCLC that originates from different cell types of origin can give rise to tumors that use distinct metastatic mechanisms (Yang *et al*, 2018). Perhaps it is thus unsurprising that SCLC is capable of evolving multiple distinct cellular mechanisms of metastasis. Existing data suggest that patients will respond differently to therapies as their tumor evolves, but it is also likely that metastatic progression and metastases in different organs may acquire unique therapeutic vulnerabilities. As new research incorporates knowledge of subtypes and specific tumor populations, additional molecular and cellular mechanisms will be identified.

### Development of new models of metastatic SCLC

The development of additional models of SCLC remains critical to further deconvolute the cellular and molecular mechanisms that drive SCLC metastasis. Current experimental systems rely on *in vitro* phenotypes associated with metastasis, *in vivo* metastases that form from xenograft transplants, and metastases that form in genetically engineered mouse models. While these models are informative, they each have limitations. For example, models of metastatic SCLC based on cell lines can potentially reflect phenotypes gained during propagation in culture, and existing genetically engineered mouse models do not yet reflect the full range and frequency of metastases in patients. While promising new models like CDXs address some of these limitations, including the development of brain metastases in some CDX models, there are still important gaps in our ability to model key aspects of SCLC metastasis. For example, studying SCLC metastases in xenograft and autochthonous mouse models is limited to metastatic tumors that develop before the mice succumb to their primary tumors. New models that overcome or circumvent these differences in frequency of metastases in specific organs will be important to address additional aspects of organ tropism of SCLC. For example, irradiating primary lung tumors at late stages in autochthonous mouse models, generating primary tumors in only one lobe followed by partial pneumonotomy, or surgically removal of subcutaneous xenografts before they grow too large may provide more time for metastases to grow in other organs.

Furthermore, many new therapies for SCLC are focused on the interactions between cancer cells and immune cells. Both adaptive and innate immune cells can play significant roles at different stages of cancer growth and the metastatic process. In other cancer types, immune responses have pro‐ and anti‐tumorigenic effects on primary tumor growth, influence dissemination, and regulate metastatic seeding and growth (Kitamura *et al*, 2015; Lambert *et al*, 2017). In SCLC, the connections between immune cells and metastases have not yet been carefully explored, but emerging evidence suggests that innate immune cells may affect the ability of SCLC cells to metastasize to the liver (Sato *et al*, 2013; Best *et al*, 2020). In this regard, it is unknown whether strategies such as inhibition of CDK4/6 kinases to protect immune cells from chemotherapy in SCLC patients (He *et al*, 2017; Lai *et al*, 2020) will have effect on metastasis. Future studies will uncover the compendium of immune cell types and interactions that influence SCLC metastases, which could uncover promising avenues for the development of therapies against SCLC metastases.

Despite decades of being understudied, SCLC may soon be leading our conceptual understanding of the determinants of metastatic ability. With a growing understanding of the impact of the cell type of origin and cancer genetics, we are poised to uncover the spectrum of cellular and molecular mechanisms that influence SCLC metastasis. The certain complexity of these mechanisms can now be embraced rather than viewed as insurmountable task. Understanding the cellular and molecular drivers of each step of SCLC development remains a formidable undertaking. While the identification of genetic and cellular heterogeneity adds multiple layers of complexity to this disease, new models are rapid enhancing our ability to study each cellular process in the metastatic cascade and connect molecular mechanisms to cellular phenotypes. Identification of cellular and molecular mechanisms that drive metastatic potential will ultimately provide targets for the development of therapies to block the development of metastatic SCLC, prevent the growth of additional metastatic tumors during and after treatment, and treat multi‐organ metastatic disease.

## Conflicts of interests

J.S. receives research funding from Stemcentrx/Abbvie and Pfizer, and licensed a patent to Forty Seven Inc./Gilead on the use of CD47 blocking strategies in SCLC. M.M.W. has equity in, and is an advisor for, D2G Oncology Inc.

**For more information**

https://www.cancer.org/cancer/lung‐cancer/if‐you‐have‐small‐cell‐lung‐cancer‐sclc.html
  A lay person description of small cell lung cancer and treatment options by the American Cancer Society.
https://www.omim.org/entry/182280
  A resource page on SCLC from the Online Mendelian Inheritance in Man online catalog of human genes and genetic disorders.
https://www.metastasis‐research.org/
  An international professional society that supports research on processes fundamental to metastasis in various cancer types.
https://depmap.org/portal/
  A database that includes a number of human SCLC cell lines and the consequences of gene knock‐down or knock‐out.
https://www.cbioportal.org/
  A portal for cancer genomics that includes human SCLC tumors.

**Pending issues**

1. Develop new pre‐clinical models that faithfully recapitulate metastasis in SCLC patients, including organ tropism.
2. Gain a better understanding of how the cell type of origin, specific genomic alterations, and cell–cell interactions influence metastatic development and response to therapy.
3. Identify therapeutic strategies that can inhibit metastatic spread in early‐stage SCLC patients and that can block metastatic growth in late‐stage SCLC patients.
